# Conserved Signaling Pathways in the *Ciona robusta* Gut

**DOI:** 10.3390/ijms25147846

**Published:** 2024-07-18

**Authors:** Marco Gerdol, Samuele Greco, Rita Marino, Annamaria Locascio, Michelina Plateroti, Maria Sirakov

**Affiliations:** 1 Department of Life Sciences, Università degli Studi di Trieste, Via Licio Giorgieri 5, 34127 Trieste, Italy; mgerdol@units.it (M.G.); samuele.greco@units.it (S.G.); 2 Department of Biology and Evolution of Marine Organisms, Stazione Zoologica Anton Dohrn, Villa Comunale, 80121 Naples, Italy; rita.marino@szn.it (R.M.); annamaria.locascio@szn.it (A.L.); 3 Institute of Genetics and Molecular and Cellular Biology, CNRS UMR7104–INSERM U1258–Université de Strasbourg, 1 Rue Laurent Fries, 67404 Illkirch, France

**Keywords:** *Ciona robusta*, gut, urochordate, RNAseq, digestive tract, signaling pathway

## Abstract

The urochordate *Ciona robusta* exhibits numerous functional and morphogenetic traits that are shared with vertebrate models. While prior investigations have identified several analogies between the gastrointestinal tract (i.e., gut) of *Ciona* and mice, the molecular mechanisms responsible for these similarities remain poorly understood. This study seeks to address this knowledge gap by investigating the transcriptional landscape of the adult stage gut. Through comparative genomics analyses, we identified several evolutionarily conserved components of signaling pathways of pivotal importance for gut development (such as WNT, Notch, and TGFβ-BMP) and further evaluated their expression in three distinct sections of the gastrointestinal tract by RNA-seq. Despite the presence of lineage-specific gene gains, losses, and often unclear orthology relationships, the investigated pathways were characterized by well-conserved molecular machinery, with most components being expressed at significant levels throughout the entire intestinal tract of *C. robusta*. We also showed significant differences in the transcriptional landscape of the stomach and intestinal tract, which were much less pronounced between the proximal and distal portions of the intestine. This study confirms that *C. robusta* is a reliable model system for comparative studies, supporting the use of ascidians as a model to study gut physiology.

## 1. Introduction

Tunicates, the sister group of vertebrates within the phylum Chordata, owe their name to the distinctive covering enveloping their bodies known as the tunic, a cellulose-containing structure unparalleled in the animal kingdom. Almost all tunicate species, including *C. robusta*, have a swimming tadpole-like larva that metamorphoses into a highly specialized juvenile, with a dramatic change in body organization [1,2]. In the adult stage, these benthic sessile filter feeders are distributed along the coastal zone, displaying remarkable resilience to pollutants. They are regarded as bioindicators of habitat conditions in coastal biotopes [3,4,5,6], thereby underscoring their growing significance in ecological and toxicological studies. Thanks to the availability of a high-quality chromosome-scale genome assembly [7], *C. robusta* (also known as *Ciona intestinalis* (Linnaeus, 1767) sp. A [8]) is a crucial model for evolutionary biology, providing important clues on early chordate evolution within the deuterostome lineage, as well as on the emergence of vertebrates [9,10]. Moreover, this tunicate is a well-established model in developmental biology because of the body plan of its larval stage, which includes prototypical important chordate features such as (i) a dorsal neural tube, (ii) an axial notochord flanked by muscle cells, and (iii) a ventral endodermal strand [11]. The low complexity of the *Ciona* gut, combined with its striking anatomical and cellular analogies to the human and murine gut [12,13,14], underscores the value of this tunicate as a model system. It is noteworthy to emphasize the increasing recognition of the extensive physiological relevance of the microbiota colonizing the gut of healthy organisms, including tunicates [15], which further strengthens the appeal of this model. The development of the digestive system of *C. robusta* takes place post-metamorphosis, as larvae lack organs specifically dedicated to food ingestion and adsorption. Consequently, all information regarding the presence and expression in the gastrointestinal tract (GI) of molecular components of important signaling pathways such as WNT, Notch, and TGFβ-BMP can be derived only from post-metamorphic or adult individuals, whereas some information is available about their role in *Ciona* at pre-metamorphic stages [16,17,18,19].

In mammals, numerous studies have explored these signaling pathways, highlighting that a complex cross-talk occurs within the intestinal epithelium. They regulate cell fate, cell proliferation, and cell death, both during development and in the adult stage, where they are also involved in tissue maintenance [20,21]. In particular, the homeostasis of the mammalian intestinal epithelium is sustained by the presence of Intestinal Stem Cells (ISCs), located within the intestinal crypts. These cells contribute to the generation of various intestinal cell types, whether under normal homeostatic conditions or in response to insults. The canonical Wnt/β-catenin pathway includes the following four main components: extracellular ligands (i.e., WNT proteins), membrane-bound receptors (i.e., FZD/LRP receptors), cytoplasmic (i.e., β-catenin, DVL, GSK-3β, AXIN, APC, CK1) and nuclear partners (activated β-catenin, which translocates to the nucleus, and TCF/LEF transcription factor family members [22]). This pathway is strongly active at the bottom of the intestinal crypts, where it is mainly implicated in controlling stemness and progenitor cell proliferation. It functions by binding Wnt and R-spondin ligands to Frizzled receptors, leading to the stabilization of β-catenin and the activation of WNT-target genes [23,24].

The Notch pathway is among the evolutionarily conserved signaling pathways that govern cell fate, cell proliferation, and cell death during development, as well as tissue homeostasis [25]. A distinctive feature of the Notch pathway lies in the transmembrane nature of both the ligand (i.e., Jag and/or Delta) and the receptor (Notch). Consequently, its signaling is constrained to neighboring cells. The basic model of Notch signaling implicates a proteolytic cleavage of the Notch protein (Adam, Serrate) to release an intracellular fragment (Nicd). In the nucleus, Nicd acts by displacing a repression complex and, together with the DNA-binding CSL (CBF1, Su(H) and LAG-1) protein, it regulates the transcription of its direct targets (Hes, Hey, etc.) [25]. Finally, the Notch pathway, in conjunction with WNT, plays a crucial role in governing stem cell identity and determining cell fate [23]. The perturbation of any of these precisely regulated signaling pathway gradients, either individually or in combination, would significantly impact the entire ISC compartment. This disruption could result in compromised tissue homeostasis and heightened susceptibility to diseases, such as cancer [21].

BMP signaling acts as an antagonist to WNT activity, as BMP and Grem1 ligands exhibit robust expression in the differentiated compartment. This creates an opposing gradient relative to WNT, thereby governing the delicate balance between epithelial differentiation and apoptosis [22,25]. The canonical TGFβ-BMP pathway is highly conserved and involves the TGFβ-BMP ligands, two cell surface BMP receptors (BMPRs), and various signal transducers (Smads) [26]. Typically, the BMP ligand triggers signaling by forming a ternary holocomplex with its receptors BMPRI and BMPRII on the cell surface. Intracellularly, Smad proteins play a pivotal role in transducing the signals from the cell surface to the nucleus.

Much of our understanding of the signaling pathways crucial to the vertebrate gut is derived from research conducted in mammalian species, including humans. However, exploring closely related outgroups to the vertebrate clade offers an opportunity to glean new insights into the evolution of the vertebrate gastrointestinal tract, shedding light on shared and divergent evolutionary traits.

In this study, we first characterized the transcriptional landscape of three distinct GI regions, i.e., the stomach (S), proximal intestine (PI), and distal intestine (DI) [27]. Then, we investigated the evolutionary conservation of WNT, Notch, and TGFβ/BMP signaling pathways along the *C. robusta* gastrointestinal tract.

## 2. Results

### 2.1. RNA-Seq Analysis and Validation of Differentially Expressed Genes (DEGs)

**Figure 1:**
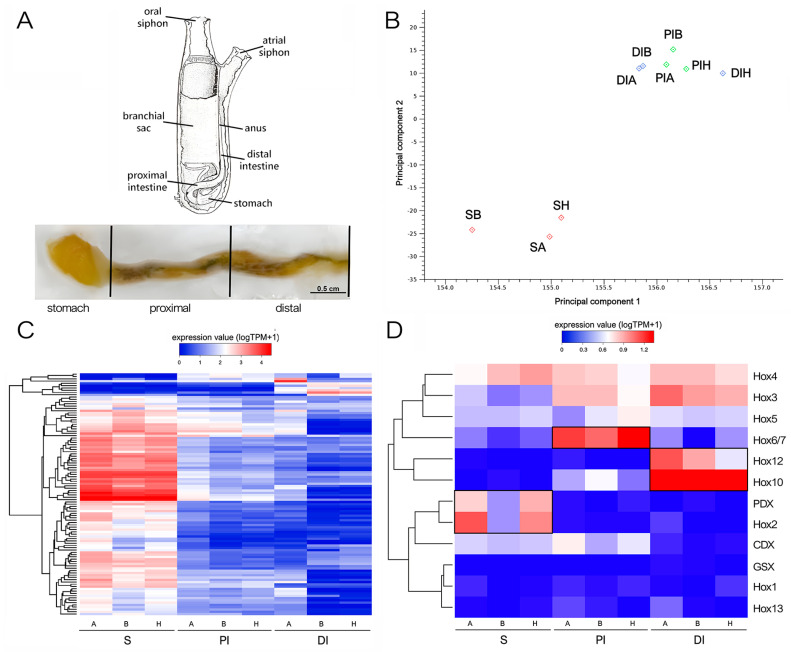
RNA-seq approach and preliminary results. (**A**) The anatomical parts of the *Ciona* gastrointestinal tract analyzed separately. S = stomach, PI = proximal intestine, DI = distal intestine, blue. (**B**) Principal component analysis, constructed based on log-transformed Transcript Per Million (TPM) expression values of all genes, summarizing the relatedness of the transcriptional profiles of the three biological samples of S (SA, SB, and SH), PI (PIA, PIB, and PIH), and DI (DIA, DIB, and DIH). (**C**) Heat map representing the log-transformed TPM expression levels of all DEGs characterized by fold change (FC) > 10 in at least one pairwise comparison among S, PI, and ID. DEGs were hierarchically clustered based on Euclidean distance, using an average linkage method. (**D**) Heat map representing the log-transformed TPM expression levels of the *Hox* and *paraHox* genes, hierarchically clustered based on Euclidean distance, using an average linkage method. Genes displaying statistically significant over-expression in one of the three GI tracts are boxed. The terms A, B, and H refer to different individual animals; for details, see the Material and Methods (Section 4).

The *C. robusta* gene expression profile was obtained by RNA-seq, which was performed on three biological replicates of the S, PI, and DI of adult ascidians (Figure 1A). The number of paired-end reads acquired through Illumina sequencing ranged from 23 M to 48 M for each sample. Both read trimming and mapping metrics highlighted a uniform quality for the reads obtained from each sample, with 0.04 ± 0.01% discarded and 81.97 ± 2.44% mapped reads per sample, respectively (Supplementary Table S2).

As shown in Figure 1B,C, the S samples displayed a markedly distinct expression profile compared with PI and DI, which showed nearly overlapping transcriptional landscapes. Differential gene expression analyses identified 290 and 204 DEGs in the S vs. PI and S vs. DI comparisons, respectively, the vast majority of which (>95%) were strongly overexpressed in S and often displayed fold change values exceeding 100 folds. By contrast, only 18 DEGs were observed in the comparison between PI and DI. This notable similarity prompted us to conduct a further analysis of DEGs by comparing S and intestine (I), without distinguishing between the two intestinal regions.

We examined the regionalized expression of *Hox* and *ParaHox* genes along the gastrointestinal tract of *C. robusta* in greater detail. Extensive literature exists on the expected expression patterns of these genes in other model species, underscoring the crucial role of tight regulation in ensuring proper morphological development [28]. This is true also for the proper development of the gastrointestinal tract in mice, chicken [29], and ascidians [14,30]. Recognizing the impact of this stringent control, even minor local fluctuations in mRNA and protein abundance can exert profound effects. Therefore, in this study, we opted for more permissive statistical thresholds to detect alterations in *Hox* and *ParaHox* gene expression levels that may hold biological significance, in contrast to the criteria applied for identifying DEGs reported in the preceding section. Specifically, we reported differential expression based on a False Discovery Rate (FDR)-corrected *p*-value threshold of 0.05.

As shown in Figure 1D, *Hox1*, *Hox13*, and *Gsx* were expressed at very low levels in all samples, never exceeding one Transcript Per Million (TPM), thus almost undetectable as reported by in situ hybridization at the juvenile stage [14]. On the other hand, the expression of *Hox3*, *Hox4*, and *Hox5* was detectable at stable levels all along the GI tract. Although *Cdx* was expressed at slightly higher levels in S and PI, the observed differences were not statistically significant. The other *Hox* genes displayed a strong regional pattern supported by high statistical significance, nearly restricted to a single segment of the GI tract. Namely, *Hox2* and *Pdx* were significantly over-expressed in S, *Hox6/7* was significantly over-expressed in PI, and *Hox 10* and *Hox12* were significantly over-expressed in DI (Figure 1D).

**Figure 2:**
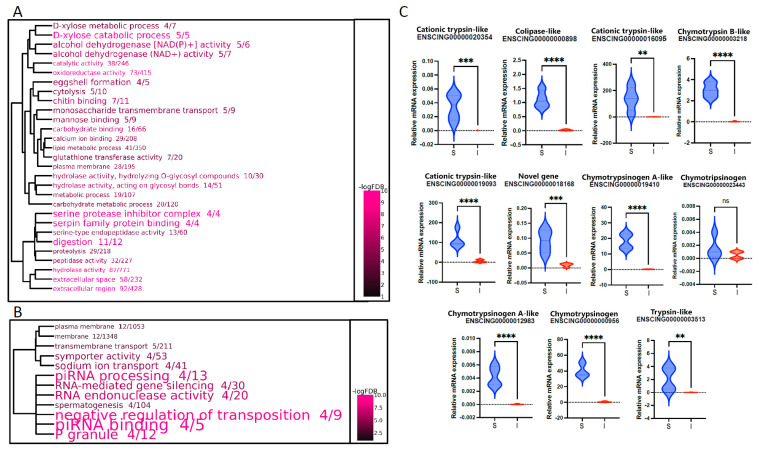
Go enrichment and validation of digestion-related genes. Dendrogram representation of the significantly enriched GO terms in the S vs. I pairwise comparison. (**A**) GO terms associated with DEGs upregulated in S. (**B**) GO terms associated with DEGs upregulated in I. The size of the GO terms indicates the observed/expected ratio, whereas the color scale represents the statistical significance. GO term clustering was based on the presence of shared DEGs. (**C**) Validation by real-time quantitative PCR (RT-qPCR) of the digestion-related genes. Violin plots represent relative expression calculated using the ΔCt method, normalization against ELF1. N = 4. ns: not significant, ** *p* < 0.01 *** *p* < 0.001, **** *p* < 0.0001.

The dendrograms reported in Figure 2A,B provide an overview of the statistical significance of the over-representation of the Gene Ontology (GO) terms associated with the 915 DEGs upregulated in S (panel A) and the 56 DEGs upregulated in I (panel B), as well as of the relationships among such annotations. Consistent with the elevated number of DEGs associated with S, this region of the digestive tract exhibited a diverse array of over-represented GO terms. The annotations specifically enriched in S were linked with digestive processes (mostly because of the strong over-expression of serine proteases and their inhibitors) and secretory hydrolases. On the other hand, the few enriched GO terms identified in I were linked with a small number of DEGs belonging to the Piwil family, which encode P-element-induced wimpy testis-like proteins and are involved in the biogenesis of piRNAs and the silencing of transposable elements.

Among the DEGs specifically expressed in the S section, for validation purposes, we focused our attention on the “digestion cluster”, which emerged among the most significantly enriched in this region because of the up-regulation of 11 out of 12 annotated genes (Figure 2A). As shown in Figure 2C, RT-qPCR confirmed the higher expression levels of these genes in S compared with the intestinal tract (i.e., PI + ID), with a statistical significance ranging from *p* ≤ 0.1 (*Cationic trypsin-like* ENSCING00000016095 and *Trypsin-like* ENSCING00000003513) up to *p* ≤ 0.001 (*Colipase-like* ENSCING00000000898, *Chymotrypsin B-like* ENSCING00000003218, *Cationic trypsin-like* ENSCING00000019093, *Chymotrypsinogen A-like* ENSCING00000019410, *Chymotrypsinogen A-like* ENSCING00000012983, *Chymotrypsinogen* ENSCING00000000956). Only the *Chymotrypsonogen* ENSCING00000023443 gene showed a trend of upregulation in S that did not reach the threshold of statistical significance.

### 2.2. Evolutionarily Conserved Signaling Pathways

The identification of orthologous genes between *Ciona* and mice, representing two lineages that have undergone more than 500 million years of independent evolution [9,31], is complicated by the frequent occurrence of lineage-specific gene duplication and loss events throughout their evolutionary history. Not surprisingly, not all the identified genes involved in the three evolutionarily conserved signaling pathways investigated in this work were found as one-to-one orthologs between the two species. Furthermore, while the automated Ensembl orthology assessments are based on both sequence homology and gene order conservation, it is important to note that the presence of orthology does not necessarily indicate functional homology between the two species. Therefore, the results presented in this section should be interpreted with caution until further functional validation is conducted.

**Figure 3:**
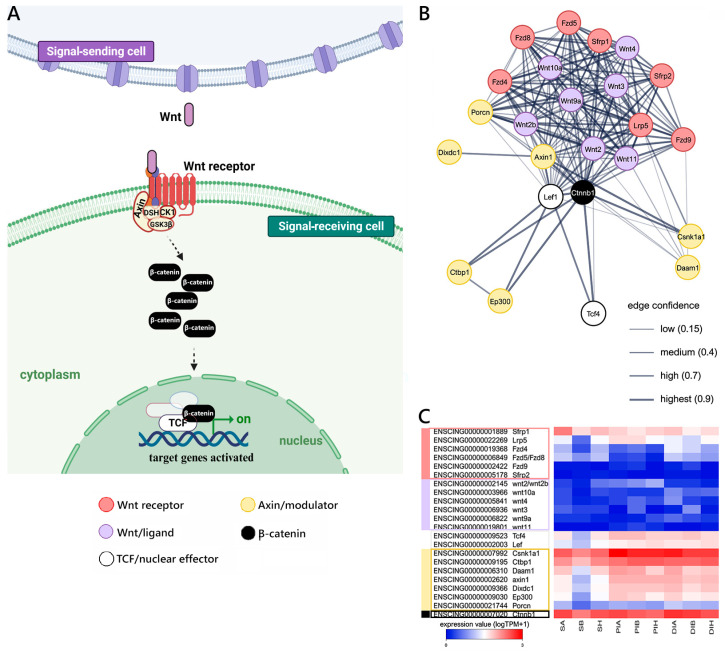
Canonical WNT signaling reconstruction. (**A**) Schematic view of the WNT/βcatenin pathway realized using BioRender.com (accessed on 13 September 2023). (**B**) STRING networks represent the interactions among selected genes linked with the Wnt pathway, arbitrarily classified as ligands, receptors, nuclear effectors, modulators, targets, and related genes. Only mouse genes sharing at least one orthologous gene with *C. robusta* are shown. Gene interaction networks were constructed based on known or inferred relationships among mouse orthologs. (**C**) Heat map representing the log-transformed TPM expression levels of the main genes (excluding target and related genes) included in the STRING network, observed in *C. robusta* S, PI, and DI samples.

Despite these shortcomings, we were able to detect the presence of several genes orthologous to well-described mouse components of the canonical WNT pathway in *C. robusta* (i.e., Wnt/β-catenin, schematized in Figure 3A). These include different Wnt ligands (i.e., *Wnt2/2b*, *Wnt3*, *Wnt4*, *wnt9a*, *Wnt10a*, *Wnt11*), Wnt receptors (i.e., *Lrp5*, *Fzd4*, *Fzd5/8*, *Fzd9*), nuclear effectors of the TCF/LEF family (*Tcf4*, *Lef*), and several modulators (*Sfrp1*, *Sfrp2*) (Figure 3B) and target and related genes (Supplementary Figure S1). As shown in the heat map, some of the most critical components of the pathway, including *Ctnnb1* (encoding β-catenin) and the nuclear effectors *Tcf* and *Lef*, were expressed at significant levels in all GI districts. On the other hand, the expression of *Wnt* ligands, albeit detectable, was invariably low in all analyzed samples, with none of the *Ciona* orthologs exceeding 6 TPM in any of the nine samples analyzed. *Wnt* receptors and modulators were generally transcribed at slightly higher levels, with *Lrp5* and *Sfrp1* in particular emerging as those characterized by the most robust expression. Interestingly, none of the major molecular players involved in the WNT pathway was differentially expressed in the comparison between different GI sections, with the exception of *Runx2* (S vs. PI) (Figure 3C and Figure S1).

**Figure 4:**
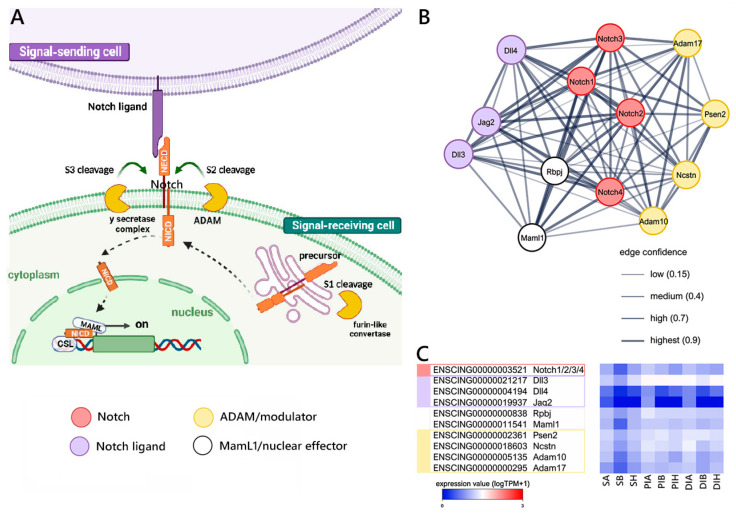
Canonical Notch signaling reconstruction. (**A**) Schematic view of the Notch pathway realized using BioRender.com (accessed on 13 September 2023). (**B**) STRING networks representing the interactions among selected genes linked with the Notch pathway, arbitrarily classified as ligands, receptors, nuclear effectors, modulators, targets, and related genes. Only mouse genes sharing at least one orthologous gene with *C. robusta* are shown. Gene interaction networks were constructed based on known or inferred relationships among mouse orthologs. (**C**) Heat map representing the log-transformed TPM expression levels of the main genes (excluding target and related genes) included in the STRING network observed in *C. robusta* S, PI, and DI samples.

Our reconstruction of the Notch signaling pathway (schematized in Figure 4A) in *Ciona* allowed for the identification of a single gene orthologous to mouse *Notch 1/2/3/4*, expressed at low but relatively stable levels along the gastrointestinal tract, that may act as a receptor. Single-copy orthologs to the mouse *Dll3*, *Dll4*, and *Jag2* ligands and the *RbpJ (Su(H))* and *Maml1* nuclear effectors were also present, together with several modulators (i.e., *Psen2*, *Ncstn*, *Adam10*, and *Adam17*) (Figure 4B), targets, and related genes (Supplementary Figure S2). As shown in Figure 4C, all the major components of the pathway were expressed along the GI tract at moderate and stable levels. However, the target gene *Fabp7* and the related gene *Runx2* displayed significant variations in expression levels between S and PI.

**Figure 5:**
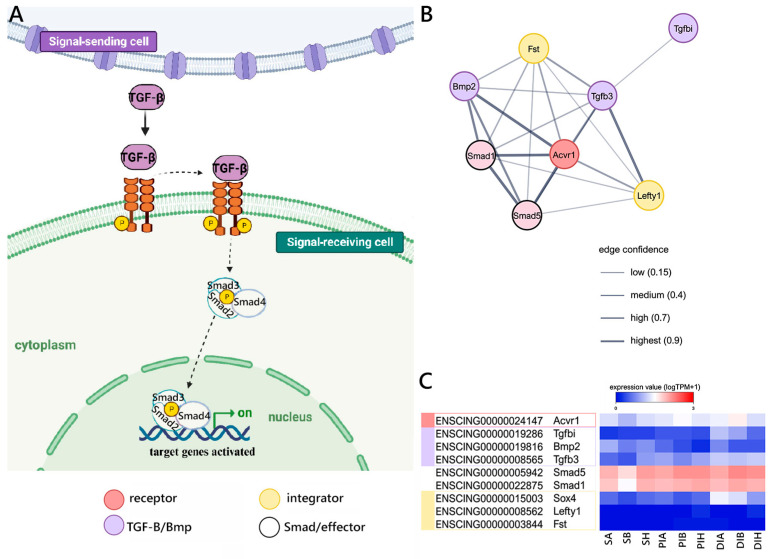
Canonical TGFβ-BMP signaling reconstruction. (**A**) Schematic view of the TGFβ-BMP pathway realized using BioRender.com (accessed on 13 September 2023). (**B**) STRING networks representing the interactions among selected genes linked with the TGFβ-BMP pathway, arbitrarily classified as ligands, receptors, nuclear effectors, modulators, targets, and related genes. Only mouse genes sharing at least one orthologous gene with *C. robusta* are shown. Gene interaction networks were constructed based on known or inferred relationships among mouse orthologs. (**C**) Heat map representing the log-transformed TPM expression levels of the main genes (excluding target and related genes) included in the STRING network, observed in *C. robusta* S, PI, and DI samples.

Finally, we investigated the components of the TGFβ-BMP signaling cascade, as schematized in Figure 5A. In detail, we were able to detect genes orthologous to the mouse for several components participating at different levels of this signaling pathway. Most notably, these included the *Acvr1* receptor, the *Bmp2*, *Tgfbi*, and *Tgfb3* ligands, the *Smad1* and *Smad5* nuclear effectors, the *Sox*, *Fst*, and *Lefty1* modulators (Figure 5B), plus a number of target and related genes (Supplementary Figure S3). The three ligands were expressed at low levels in all GI tracts, with *Tgfb3*, in particular, never exceeding 1 TPM and therefore unlikely to have a relevant biological role in this context. On the other hand, both the *Acvr1* receptor, the *Smad1* and *Smad5* nuclear receptors, and the *Sox* modulator were expressed at stable levels along the GI tract. The two other modulators, i.e., *Fst* and *Lefty1*, were expressed at negligible levels (<1 TPM) in all samples (Figure 5C).

## 3. Discussion

The primary aim of this study was to assess the suitability of *Ciona* as a model for investigating gut physiopathology. To accomplish this objective, we investigated the presence of evolutionarily conserved genes belonging to signaling pathways of pivotal importance for gut development and investigated the transcriptional profiles of three distinct regions of the *Ciona* gastrointestinal tract, namely, the stomach and the proximal and distal intestines.

The lack of any differential expression of the genes involved in digestive processes and the high similarity between the PI and DI profiles indicate that there is no regionalization for the digestion of the different nutrients.

On the other hand, the regional expression of some *Hox* (i.e., *Hox2*, *Hox6/7*, *Hox10*, and *Hox12*) and *Para-Hox* (*Pdx*) genes along the gastrointestinal anterior–posterior axis contributes to strengthening the hypothesis of specific physical compartmentalization. Although the data reported in this study derive from adult individuals, these observations are consistent with data previously collected in the juvenile stages of *C. robusta* and its congeneric species *C. intestinalis* and *C. savigny* by RNA-seq or in situ hybridization [12,13,14,30,32].

The Wnt/β-catenin, Notch, and TGFβ-BMP signaling pathways characterize the vertebrate gastrointestinal tract, playing a crucial role in gut development and homeostasis. These pathways act individually or engage in cross-talk, contributing to various aspects of mammalian intestinal physio-pathology [33,34]. For instance, Wnt and Notch pathways control stem cell biology and cell lineage differentiation, while TGFβ/BMP is involved in sustaining a differentiated phenotype in the vertebrate GI tract.

This is the first effort to explore the presence and delineate the expression patterns of the molecular components associated with the Wnt/β-catenin, Notch, and TGFβ-BMP signaling pathways within the gastrointestinal tract of adult *Ciona* organisms. Prior to this work, the activity of the Notch pathway was only reported in the context of oral siphon regeneration in adult *Ciona* individuals [18]. On the other hand, while descriptions of the Wnt/β-catenin and TGFβ-BMP signaling pathways were previously provided, their specific functions were primarily analyzed in the context of developmental processes [16,17,35].

Our observations in the context of the gastrointestinal system not only align with the pivotal phylogenetic position of tunicates as the sister group of vertebrates in chordate evolution [9,10,36,37] but also lay the first stone towards a novel use of *C. robusta* as a model for comparative physiology with vertebrates and humans. Finally, these results may pave the way for the use of *Ciona* as a model to investigate different aspects of intestinal physio-pathology, including the response to ingested pollutants and emerging contaminants.

## 4. Materials and Methods

### 4.1. Samples Collection

Adult animals were purchased from Il Cozzaro Nero s.r.l. (Taranto, Italy) from the Mar Piccolo basin in Taranto (Southern Italy), brought to the SZN animal facility alive and starved 48 h prior to dissection. All procedures were in compliance with the current regulations for the experimental use of live invertebrate animals. For each animal, we collected the stomach (S) and divided the intestine into two parts as follows: the proximal intestine (PI), extending from the end of the stomach up to the first anatomical band, and the distal intestine (DI), corresponding to the following portion of intestine without the anus, according to Millar’s description of the adult *Ciona* gastrointestinal tract [27] (Figure 1A). All the samples were frozen with liquid nitrogen and stored at −80° until RNA extraction.

### 4.2. RNA-Seq

Total RNA was extracted from the biological samples described in the previous section, obtained from eight different animals, by using a TRIZOL© (Termo Fisher Scientific, Santa Clara, CA, USA). Following homogenization, all samples were subjected to RNA extraction and purification according to the manufacturer’s instructions. An initial assessment of the quality of extracted RNA, carried out with an Agilent bioanalyzer 2100 system (Agilent Technologies, Santa Clara, CA, USA), led to the selection of three individuals that satisfied high-quality criteria, i.e., Optical Density (OD) 260/280 = 1.96–2.06, OD 260/230 ≥ 1.9, RNA Integrity Number > 8.5, 28/18S ≥ 1.1 in all the three GI tracts. These samples, which represented the three biological replicates for each respective region (namely, “SA”, “PIA”, and ”DIA” from animal “A”; “SB”, “PIB”, and “DIB” from animal “B”; and “SH”, “PIH”, and “DIH” from animal “H”), were subject to RNA-sequencing. Construction of cDNA libraries and sequencing were performed by Genomix4life SRL using a TruSeq stranded mRNA library preparation kit and an Illumina NextSeq 550 platform, with the aim to obtain approximately 20 million paired-end (PE) reads per sample, using a 2 × 75 cycles sequencing strategy. Raw reads were deposited in the NCBI SRA database under the BioProject accession ID PRJNA1031967.

### 4.3. Bioinformatic Analyses

Raw Illumina reads were imported into the CLC Genomics Workbench v. 23 environment (Qiagen, Hilden, Germany) and underwent a strict trimming procedure, which detected and removed sequencing adapters and low-quality bases (identified based on a quality threshold = 0.05) and discarded the first 12 nucleotides at the 5’end of each read that were affected by compositional bias. Finally, residual reads shorter than 40 nt were discarded, and only intact read pairs were considered for further analysis.

Trimmed reads were mapped against the KH reference genome (version GCF_000224145.3) using the Ensembl annotation, as in preliminary tests it led to higher mapping rates compared with the alternative available options (i.e., the NCBI RefSeq and ANISEED annotations). Mapping was carried out with the *RNA-seq analysis* tool using stringent alignment criteria (i.e., length fraction = 0.75, similarity fraction = 0.98). Total exon read counts were used to compute gene expression levels for each annotated gene using the TPM metric, which ensures comparability both within and between samples [38].

Principal Component Analyses and heat maps, constructed through hierarchical clustering of samples using log-transformed TPM values, were employed for the initial evaluation of consistency among biological replicates, ruling out the presence of outlier samples. DEGs in all possible pairwise comparisons (i.e., S vs. PI, S vs. DI, and PI vs. DI) were identified with EDGEr [39], based on combined FDR-corrected *p*-value (<1 × 10^−5^) and fold change (FC, ±2) thresholds. To graphically summarize the main differences among tissues, the expression levels of a subset of DEGs displaying high tissue-specificity (i.e., FC values >10 in at least one pairwise comparison) were log-transformed and plotted in a heat map, which hierarchically clustered DEGs based on Euclidean distance using an average linkage method. Because of the nearly overlapping gene expression profiles obtained from PI and DI, an additional differential gene expression analysis was run, using the same parameters described above, to detect DEGs in the S vs. I comparison (i.e., considering both PI and DI samples as part of the same group). The resulting DEGs were subjected to Gene Set Enrichment Analysis (GSEA) [40] through a hypergeometric test run on the associated GO terms. Significant over-representation was considered for FDR-corrected *p*-values < 0.05 and observed–expected values > 3. The results of this functional enrichment analysis were graphically represented with dendrograms, using a modified version of GO_MWU [41], as previously described by Greco and colleagues [42].

The orthology relationship between *C. robusta* and mouse genes was extracted from Ensembl, which makes such assignments based on two combined independent lines of evidence, i.e., whole-genome alignment and gene order conservation [43]. This enabled the creation of three distinct gene lists based on the literature data [44], corresponding to the WNT, Notch, and TGFβ/Bmp pathways. These lists were used as inputs for the generation of gene interaction networks with STRING v.11 [45], which exploited both known (i.e., experimentally determined) and predicted interactions.

### 4.4. Real-Time Quantitative PCR (RT-qPCR)

Validation of selected DEGs was performed by RT-qPCR using samples from four different animals. The first strand cDNA was synthesized using 1 µg total RNA per 20 µL reaction system by reverse transcriptase (Vazyme Biotech, Nanjing, China) following the manufacturer’s protocol and stored at −20 °C. Primer design was implemented by aligning de novo assembled transcripts sequenced with the reference genome with Splign [46] in order to verify the correctness of annotated exons and design primers within adjacent exons. RT-qPCR assays were performed in triplicate using the QuantStudio™ 5 Real-Time PCR System (Applied Biosystems, Waltham, MA, USA). Each assay included a no-template control for each primer pair. The relative expression level of each DEG was calculated by applying the ΔCt method [47], considering E = 2 and using *ENSCING00000012434* (*elongation factor 1-beta-like*, *ELF1*) as a reference gene for normalization. The primer sequences are reported in Supplementary Table S1.

### 4.5. Statistical Analysis

Statistical analyses of data represented in graphs were conducted using GraphPad Prism software (version 10; GraphPad Software Inc., San Diego, CA, USA). The Student *t*-test (unpaired, 2-tailed) was performed to analyze the statistical significance between groups, and the level of significance was established as a *p*-value < 0.05.

## Data Availability

All the data analyzed in this study were deposited in the NCBI SRA database under the BioProject accession ID PRJNA1031967.

## Supplementary Materials

The following supporting information can be downloaded at: https://www.mdpi.com/article/10.3390/ijms25147846/s1.
