# Supplementary material for: Conserved Signaling Pathways in the Ciona robusta Gut
Source: Int J Mol Sci. 2024 Jul 18;25(14):7846. doi: 10.3390/ijms25147846 (PMC11277035; doi:10.3390/ijms25147846)
Supplement: Supplementary file 1 [file ijms-25-07846-s001.zip › ijms-3065153-supplementary.pdf]

Supplementary Figures and Tables

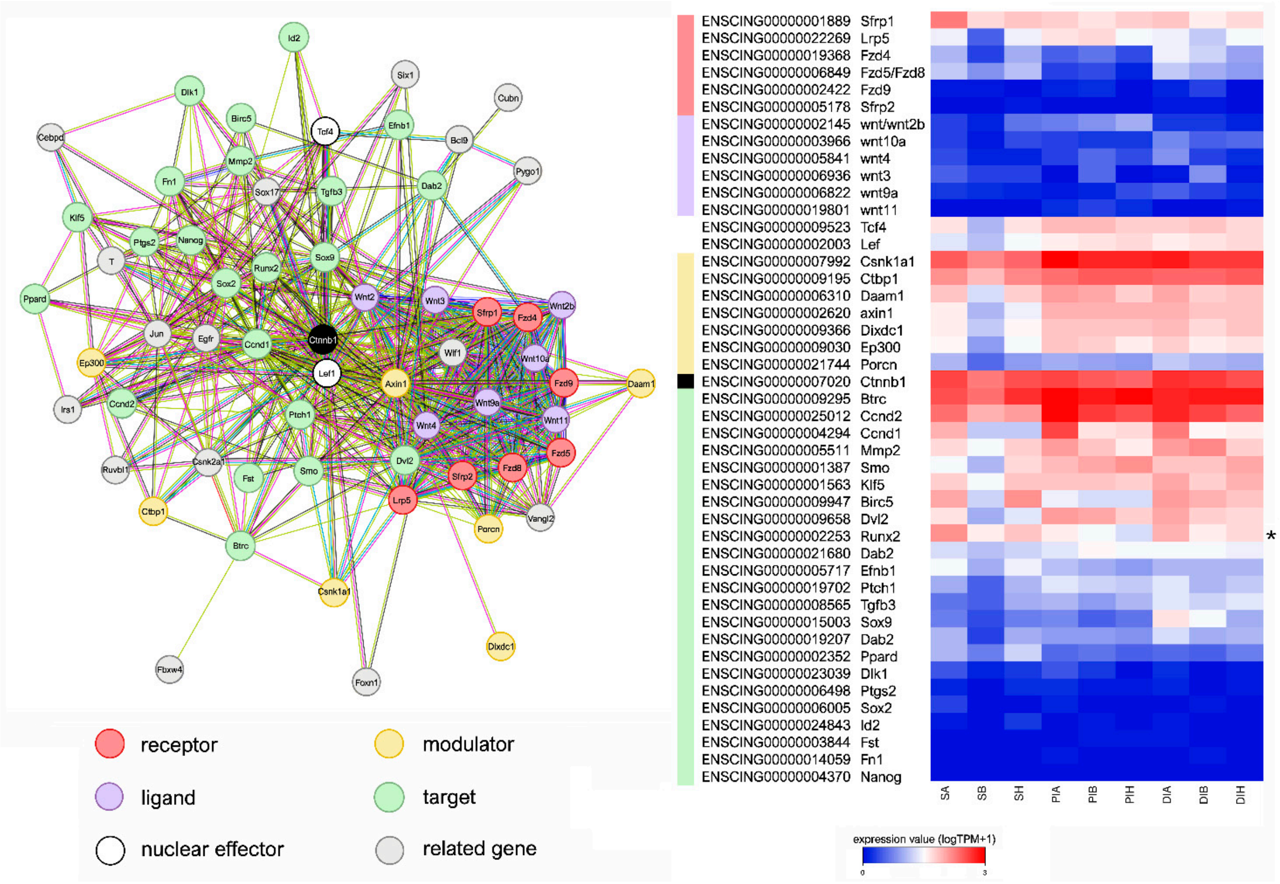

Supplementary Figure S1. (Left panel) STRING networks representing the interactions between selected genes linked with the Wnt pathway. Only mouse genes sharing at least one orthologous gene in *C. robusta* are shown. Gene interaction networks were constructed based on known or inferred relationships among mouse orthologs. *Ctnnb1* is represented in black, whereas according to the legend the other genes are arbitrarily classified as ligands (violet), receptors (red), nuclear effectors (white), modulators (yellow), targets (green) and related genes (Grey). (Right panel) Heat map representing the log-transformed TPM expression levels of the main genes (excluding target and related genes) included in the STRING network, observed in *C. robusta* S, PI and DI samples. Genes expressed at low, medium and high levels are marked in blue, white and red, respectively. An asterisk marks the statistically significant differential overexpression of Runx2 in the S vs PI comparison.

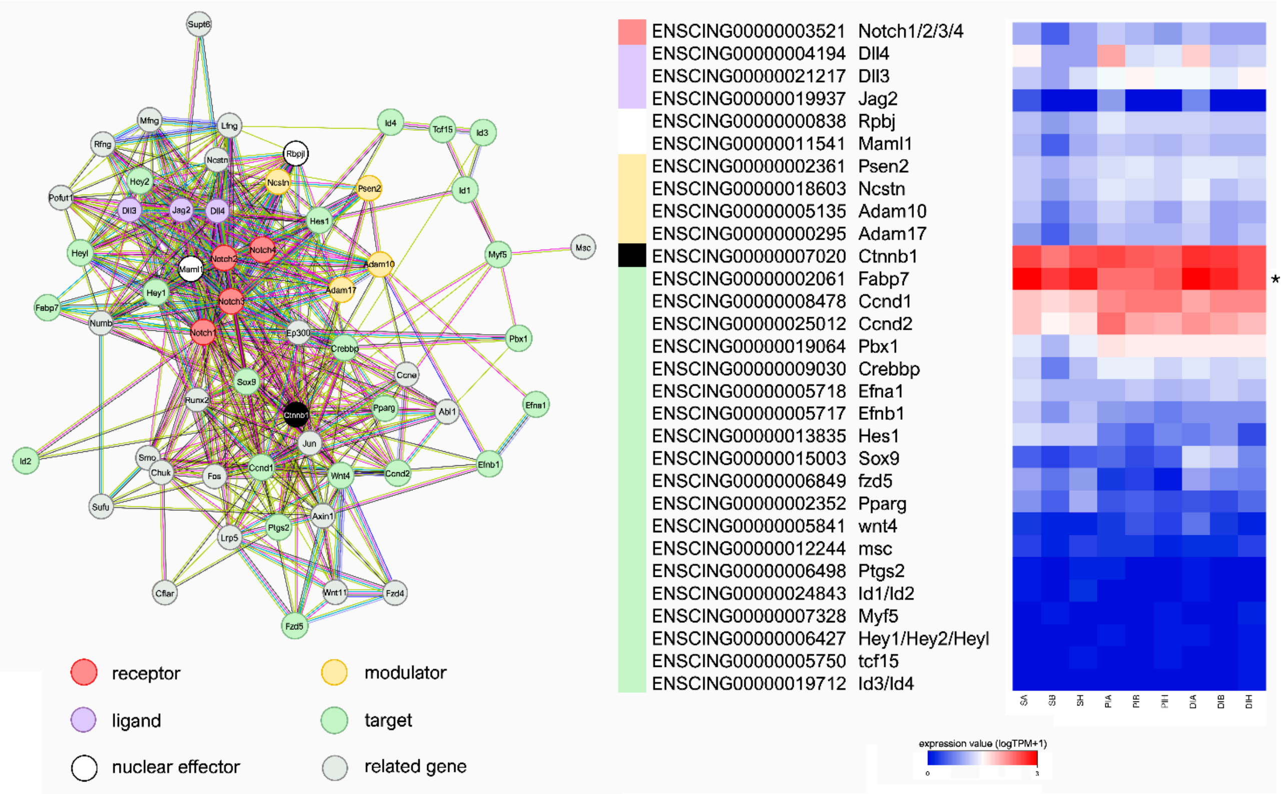

Supplementary Figure S2. (Left panel) STRING networks representing the interactions between selected genes linked with the Notch pathway. Only mouse genes sharing at least one orthologous gene in *C. robusta* are shown. Gene interaction networks were constructed based on known or inferred relationships among mouse orthologs. *Ctnnb1* is represented in black, whereas according to the legend the other genes are arbitrarily classified as ligands (violet), receptors (red), nuclear effectors (white), modulators (yellow), targets (green) and related genes (Grey). (Right panel) Heat map representing the log-transformed TPM expression levels of the main genes (excluding target and related genes) included in the STRING network, observed in *C. robusta* S, PI and DI samples. Genes expressed at low, medium and high levels are marked in blue, white and red, respectively. An asterisk marks the statistically significant differential overexpression of Fabp7 in the S vs PI comparison.

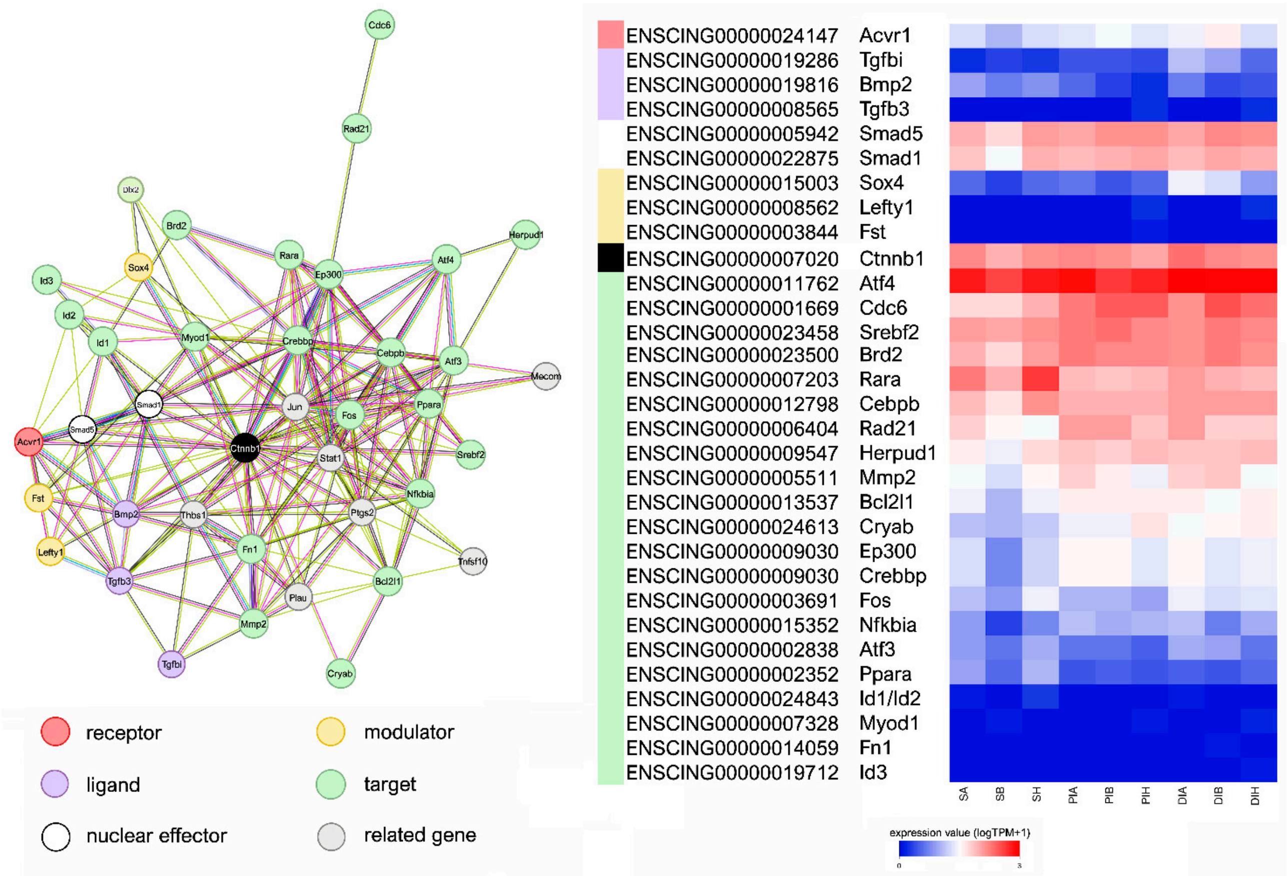

Supplementary Figure S3. (Left panel) STRING networks representing the interactions between selected genes linked with the BMP/TGFβ pathway. Only mouse genes sharing at least one orthologous gene in *C. robusta* are shown. Gene interaction networks were constructed based on known or inferred relationships among mouse orthologs. *Ctnnb1* is represented in black, whereas according to the legend the other genes are arbitrarily classified as ligands (violet), receptors (red), nuclear effectors (white), modulators (yellow), targets (green) and related genes (Grey). (Right panel) Heat map representing the log-transformed TPM expression levels of the main genes (excluding target and related genes) included in the STRING network, observed in *C. robusta* S, PI and DI samples. Genes expressed at low, medium and high levels are marked in blue, white and red, respectively.

sample	number of raw reads	number of trimmed reads	discarded reads	mapping rate
SA	42929530	42919511	0,02%	84,18%
SB	23083962	23078460	0,02%	78,64%
SH	36842296	36833692	0,02%	78,20%
PIA	36983066	36966614	0,04%	83,73%
PIB	38718538	38701540	0,04%	80,34%
PIH	41391282	41372395	0,05%	84,16%
DIA	48495928	48474177	0,04%	84,57%
DIB	36379196	36363040	0,04%	82,16%
DIH	47838100	47816443	0,05%	81,74%

Supplementary Table S1. Summary of sequencing outputs, trimming and mapping rates for the nine samples analyzed in this study.

Ensembl gene ID		Primer sequence 5′-3′	Amplicon size	Description
ENSCING00000012434	F	AGAGAAGACCGACTTGCTGC	100bp	***Elongation factor 1-beta-like (Elf1)***
	R	CGTCTCCCCACGGTTTAACA		
ENSCING00000003218	F	TACCCTATACGCCCCCACAA	116bp	***Chymotrypsin B-like***
	R	TACCATTGGCTGGTACGCTG		
ENSCING00000023443	F	CGCTGGAGGAGCAAACCTTA	133bp	***Chymotripsinogen***
	R	TGGGTAAGATGTACCGCAGC		
ENSCING00000020354	F	GCAGCAGCTGGTAACTATGAC	123bp	***Cationic trypsin-like***
	R	CTCCGCACATAAGGCTTCCA		
ENSCING00000003513	F	CGGTCAAGATTCCTACGATGGTG	103bp	***Trypsin-like***
	R	AACTGGGCCACCAGAATCAC		
ENSCING00000019410	F	ACAAGAATACGCCGCTGGAA	131bp	***Chymotrypsinogen A-like***
	R	CTCTGCAGTCTGCTACGGAA		
ENSCING00000019093	F	TTCCCTTACTGTTACTGCTGGAGAC	210bp	***Cationic trypsin-like***
	R	GAGTTCTGGCTGATGCTGCTTG		
ENSCING00000012983	F	AGCTCTGGCTTCGGATACAC	113bp	***Chymotrypsinogen A-like***
	R	GACCAAGGCTGGATTGCTGA		
ENSCING00000000956	F	CCATGGCAAGTTTCCCTCCA	119bp	***Chymotripsinogen***
	R	ACAGTTGTGCTGGACGCTAT		
ENSCING00000000898	F	TCCTAAAAGTAGACAGCTAAAATG	142bp	***Colipase-like***
	R	ATTGATCGCCTTCGACGCAA		
ENSCING00000016095	F	GCCGTAGGAACTGTCTGCAAAG	122bp	***Cationic trypsin-like***
	R	GCGTTGTTGCAGTCAGAGTTGG		
ENSCING00000018168	F	CTGTTGTACTCGGCGACCATG	152bp	***Novel gene***
	R	GTTTAACACGGCGGGAGTCG		

Supplementary Table S2. RT-qPCR Primers sequence and amplicon size.
